# Trends in incidence and detection of advanced breast cancer at biennial screening mammography in The Netherlands: a population based study

**DOI:** 10.1186/bcr3091

**Published:** 2012-01-09

**Authors:** Joost Nederend, Lucien EM Duijm, Adri C Voogd, Johanna H Groenewoud, Frits H Jansen, Marieke WJ Louwman

**Affiliations:** 1 Department of Radiology, Catharina Hospital, PO Box 1350, 5602 ZA, Eindhoven, The Netherlands; 2 Department of Epidemiology, Maastricht University, PO Box 616, 6200 MD, Maastricht, The Netherlands; 3 Comprehensive Cancer Centre South (IKZ)/Eindhoven Cancer Registry, PO Box 231, 5600 AE, Eindhoven, The Netherlands; 4 Expertise Center Transitions of Care, Rotterdam University of Applied Sciences, PO Box 25035, 3001 HA, Rotterdam, The Netherlands

## Abstract

**Introduction:**

The aims of this study were to determine trends in the incidence of advanced breast cancer at screening mammography and the potential of screening to reduce it.

**Methods:**

We included a consecutive series of 351,009 screening mammograms of 85,274 women aged 50-75 years, who underwent biennial screening at a Dutch breast screening region in the period 1997-2008. Two screening radiologists reviewed the screening mammograms of all advanced screen detected and advanced interval cancers and determined whether the advanced cancer (tumor > 20 mm and/or lymph node positive tumor) had been visible at a previous screen. Interval cancers were breast cancers diagnosed in women after a negative screening examination (defined as no recommendation for referral) and before any subsequent screen. Patient and tumor characteristics were compared between women with advanced cancer and women with non-advanced cancer, including ductal carcinoma in situ.

**Results:**

A total of 1,771 screen detected cancers and 669 interval cancers were diagnosed in 2,440 women. Rates of advanced cancer remained stable over the 12-year period; the incidence of advanced screen-detected cancers fluctuated between 1.5 - 1.9 per 1,000 screened women (mean 1.6 per 1,000) and of advanced interval cancers between 0.8 - 1.6 per 1,000 screened women (mean 1.2 per 1,000). Of the 570 advanced screen-detected cancers, 106 (18.6%) were detected at initial screening; 265 (46.5%) cancers detected at subsequent screening had been radiologically occult at the previous screening mammogram, 88 (15.4%) had shown a minimal sign, and 111 (19.5%) had been missed. Corresponding figures for advanced interval cancers were 50.9% (216/424), 24.3% (103/424) and 25.1% (105/424), respectively. At multivariate analysis, women with a ≥ 30 months interval between the latest two screens had an increased risk of screen-detected advanced breast cancer (OR 1.63, 95%CI: 1.07-2.48) and hormone replacement therapy increased the risk of advanced disease among interval cancers (OR 3.04, 95%CI: 1.22-7.53).

**Conclusion:**

We observed no decline in the risk of advanced breast cancer during 12 years of biennial screening mammography. The majority of these cancers could not have been prevented through earlier detection at screening.

## Introduction

Most breast cancer deaths are due to advanced disease, diagnosed when it has already spread to lymph nodes or distant organs. Therefore, many countries have introduced breast cancer screening programs in order to detect breast cancer at an early stage. In the Netherlands, a nation-wide biennial screening program for women aged 50-69 years was implemented between 1989 and 1997. In 1998, the upper age limit for breast screening was extended to 75 years. The attendance rate at our screening region is 84% [1].

Several studies conclude that screening mammography is effective in reducing breast cancer mortality [2-4]. However, the authors of a recent comprehensive review stated that the positive results of randomized trials of mammography screening on breast cancer mortality should be interpreted with caution as these trials were carried out in an era before the use of anti-hormonal therapies and before major advances in other aspects of breast cancer treatment were made [5]. Autier et al. compared breast cancer mortality in 30 European countries and concluded that the reduction in breast cancer mortality was more profound in non-screened women (-37%) than in screened women (-21%) [6]. It remains a question of debate which part of the reduction can be attributed to screening and which part can be explained by other factors, such as the more extensive use of adjuvant systemic treatment [7,8]. Compared with rates in 1986-1988, Otto et al. reported a 19.9% reduction in breast cancer mortality rate in 2001 as a result of routine mammography screening in the Netherlands [4]. Kalager et al. calculated a non-significant, 10% mortality reduction in screened versus non-screened areas in Norway [8]. Jørgensen et al. did not find any effect of breast cancer screening on breast cancer mortality in Denmark and they contributed the lower mortality to changes in risk factors and improved treatment [9].

If a mortality reduction is due to screening rather than the result of adjuvant systemic therapy, one would expect that it is preceded by a decrease in risk of a diagnosis of advanced breast cancer. Screening may not be that effective if no stage shift is observed. Although Fracheboud et al. initially found a significant decrease in the incidence rate of advanced disease in women who participated in the Dutch screening program, they later reported an increase in advanced cancers detected at screening [10,11]. For more recent years of nation-wide screening, the National Evaluation Team for Breast Cancer Screening in The Netherlands found a more or less stable tumor size distribution of screen-detected cancers, as well as a stable rate of lymph node positive breast cancers [12]. Autier et al. [13] observed no significant changes in advanced breast cancer rates in several European countries, despite good participation at screening mammography programs.

So far, no data are available for predictors for a diagnosis of advanced disease. The primary goal of our population based study was to determine the trends in incidence and detection of advanced breast cancer in the Southern Region of the nationwide breast screening program in the Netherlands, during twelve years of biennial screening mammography. We also assessed the proportion of advanced cancers that potentially could have been prevented through earlier detection at screening and we identified patient and tumor characteristics that were related to an increased risk of advanced breast cancer.

## Materials and methods

### Study population

We included 351,009 consecutive screens (46,155 initial screens and 304,854 subsequent screens) of 85,274 women, who underwent biennial screening mammography at two specialized analogue screening units in the Southern Region (BOBZ, Bevolkings Onderzoek Borstkanker Zuid) of the Dutch nationwide breast cancer screening program between January 1, 1997 and January 1, 2009. Prior to a screening examination women are asked whether their screening and follow-up data can be used for evaluation purposes. Only three women had not given this written informed consent and they were not included in the study population. Ethical approval for this study was waived by the Central Committee on Research Involving Human Subjects (CCMO) in The Hague, The Netherlands.

### Screening procedure and referral

Details of our nation-wide breast cancer screening program, offering biennial screening mammography for women aged 50-75 years, are described elsewhere [14,15]. In brief, all mammograms were obtained by specialized screening mammography technologists and independently double read by certified screening radiologists. In the Southern Region, technologists have been actively participating in the assessment of screening mammograms, in addition to the double reading by the radiologists [16]. Fifteen certified screening radiologists were involved, all of whom evaluated at least 5,000 screening mammograms yearly. Prior screening mammograms were always available for comparison at the time of subsequent screening. Women were asked to fill in a questionnaire prior to screening mammography with questions about date, type and reason of previous breast surgery, family history of breast cancer and hormonal replacement therapy. For all women with a positive screening mammogram or interval cancer, we recorded the information of this questionnaire in a database which is used for quality assurance of our screening program. We consulted the clinical records of the hospital to which a woman had been referred for the completion of missing data (62 women). If screening mammography showed a suspicious or malignant lesion, the woman was referred to a surgical oncologist or breast clinic for further analysis of the mammographic abnormality.

### Workup facilities at hospitals

A total of 16 hospitals in the Southern Region were involved in the diagnostic workup, of which four centrally located hospitals accounted for the workup of 93% (4,137/4,450) of referred women [17]. These four hospitals performed between 2,000-3,500 diagnostic mammographic examinations yearly. Further evaluation depended on the workup protocols and facilities available, and consisted of additional mammographic views, breast ultrasound, magnetic resonance mammography, percutaneous fine needle aspiration or core biopsy (usually image guided), or open surgical biopsy. Out-patient breast clinics were introduced between 1999 and 2007 and between 2002 and 2007 hospitals implemented multidisciplinary teams (including surgical oncologists, radiologists, clinical oncologists, pathologists and dedicated breast nurses or physician assistants) for a systematic discussion of the clinical, radiologic and biopsy findings of each referred woman. New diagnostic techniques were also introduced over time, including Magnetic Resonance Mammography (2000-2004), 14-Gauge stereotactic core needle biopsy (2000-2007), axillary ultrasound with lymph node sampling (1998-2000), and 9- or 10-Gauge stereotactic vacuum-assisted core biopsy (2004-2007). One hospital mainly performed ultrasound guided fine needle aspiration cytology of solid breast lesions, whereas the other three hospitals gradually replaced cytology by 14-18 Gauge core biopsies.

### Follow-up procedure

During a follow-up period of two years, we collected data on diagnostic and surgical procedures, histopathology and TNM (tumor-node-metastasis) classification [18] of all screen detected cancers and interval cancers. Interval cancers were defined as breast cancers diagnosed in women after a screening examination yielded negative results (defined as no recommendation for referral) and before a subsequent biennial screen was performed. Procedures for the detection of interval cancers have been described previously [19].

Breast cancers were divided into ductal carcinoma in-situ and invasive cancers; lobular carcinoma in-situ was considered to be a benign lesion. Ductal carcinoma in situ was included in the group of non-advanced breast cancers. Advanced cancers were defined as cancers with TNM stage IIA or higher, i.e. tumor size exceeding 20 mm (T2) and/or presence of lymphatic metastasis in the sentinel node or axillary lymph nodes. Sentinel nodes were classified negative if they harbored isolated tumor cells or sub-micrometastases (< 0.2 mm) and were considered positive (N+) if they contained micrometastases (0.2-2 mm) or macrometastases (> 2 mm). A further subdivision of advanced cancers in T1N+, T2+N- and T2+N+ was also made to be able to determine a possible effect of the introduction of sentinel node biopsy and concomitant stage migration on our findings and we analyzed our data using different definitions (T1N+, T2+N- or T2+N+) of advanced cancer.

Incidence rates of advanced cancers in the Southeastern Netherlands between 1980 and 2009 were calculated for all women (whether screened or not) using the population-based Eindhoven Cancer Registry [20].

Invitation letters for screening mammography are routinely sent 23-26 months after the previous screening round. If a woman is not able to attend screening, she is given the opportunity to make a new appointment within 6 months. Screening intervals exceeding 30 months usually involve women who have missed one or more screening rounds. Therefore, we considered a screening interval of more than 30 months to be a prolonged screening interval. For each woman with a screen detected cancer and a screening interval of more than 30 months prior to the latest screening examination, we determined if she had undergone clinical mammography at any of the hospitals located at our screening region within 30 months prior to final screening. If the latter was the case (n = 6), then the woman was considered to have a screen interval of less than 30 months.

### Review of screening mammograms of advanced breast cancers

Two experienced screening radiologists (LD, FJ) reviewed the two most recent screening mammograms of all women with advanced screen detected breast cancers at a subsequent screen. Older screening examinations were available for comparison if desired by the radiologists. They determined whether or not the cancer had been missed, had shown a minimal sign [21] or had been occult at the previous screen. For each advanced interval cancer, the radiologists correlated the clinical mammogram, on which the interval cancer had been diagnosed, with the latest screening examination and also determined whether the cancer had been visible at the latest screen. The radiologists classified the mammographic abnormality of each advanced breast cancer into one of the following, mutually exclusive, categories: 1. suspicious high density (e.g., spiculated density or density with indistinct borders); 2. suspicious microcalcifications (e.g., pleomorphic, branching, or amorphous/indistinct microcalcifications); 3. high density in combination with microcalcifications; 4. architectural distortion or 5. breast parenchyma asymmetry. Finally, the breast density of the latest screen (and of the last but one screen in case of subsequent screening) was assessed, according to the BI-RADS criteria [22]. The radiologists were initially blinded to each other's review and discrepant readings were followed by consensus reading.

### Statistical analysis

Statistical analyses were performed per 2-year screening periods. All data were entered into a computerized spreadsheet (Excel; Microsoft, Redmond, WA, USA). Statistics were performed using the SAS program version 9.1.3 (Statistical Analysis Software; SAS/STAT software^®^, Cary, NC, USA). A double sided t-test was used to test differences between continuous variables, and the χ^2 ^test to test differences between categorical variables. Logistic regression was performed to investigate which factors significantly affected the risk of a diagnosis of advanced breast cancer among patients with screen-detected and interval breast cancer. The significance level was set at 5%.

## Results

### Overall screening results

**Figure 1:**
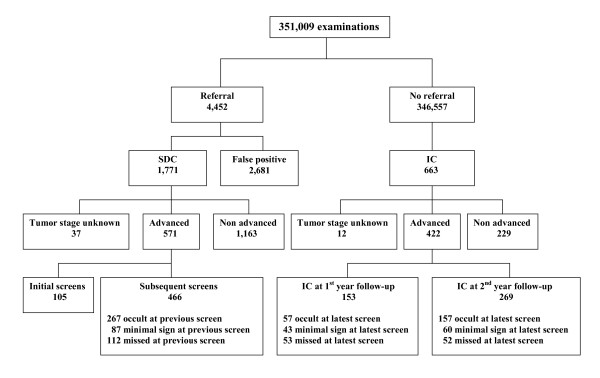
**Mammography screening outcome at 2-year follow-up**. SDC = screen-detected cancer; IC = interval cancer.

**Figure 2:**
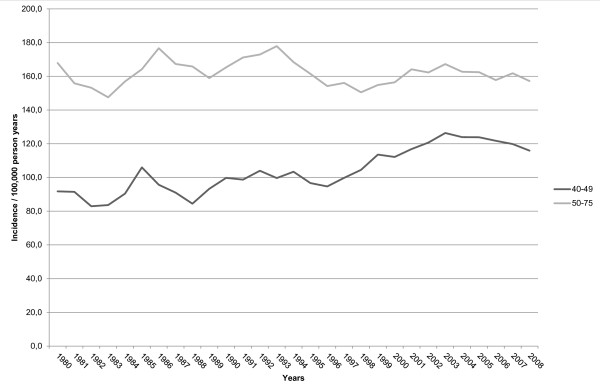
**Time trend in incidence of advanced breast cancer among women aged 40-49 years and 50-75 years in southeastern Netherlands, 1980-2009**.

The biennial number of screening examinations gradually increased from 48,721 (1997/1998) to 67,530 (2007/2008) and the biennial referral rate varied between 0.9% and 1.6% (mean 1.3%). Breast cancer was diagnosed in 1,771 of 4,450 referred women (including 287 ductal carcinomas in situ), resulting in an overall cancer detection rate of 5.1 per 1,000 screens and an overall positive predictive value of 39.8% (Table 1 Figure 1). In addition, 669 interval cancers (including 27 ductal carcinomas in situ) were diagnosed. Mean sensitivity of breast cancer screening was 72.6% (1,771/2,440). Screening sensitivity was higher for non-dense breasts (ACR breast density category I+II), that is, 75.3% (1,189/1,580), than for dense breasts (ACR breast density category III+IV), that is, 67.7% (582/860) (p < 0.001). The proportion of advanced cancers among all cancers was 40.7% (994/2,440). This proportion did not change significantly through the years and ranged from 37.8% (1997/1998) to 45.5% (2001/2002) (p = 0.6, Table 1). Visibility of screen detected cancers and interval cancers on previous screening rounds remained constant during the twelve year screening period and the proportion of occult cancers, minimal signs and missed cancers for both groups neither changed (p = 0.4 and p = 0.5, respectively). Figure 2 shows that advanced breast cancer rates in the screened age group were stable between 1980-2009 and showed no decline after the introduction of screening mammography in southeastern Netherlands. It also shows a gradual increase in advanced breast cancer rate in women less than 50 years of age.

**Table 1:** Screening results at 6 consecutive 2-year screening periods

Screening period	1997/1998	1999/2000	2001/2002	2003/2004	2005/2006	2007/2008	Total
							
Mammograms, No	48,721	53,718	53,489	61,251	66,300	67,530	351,009
Referral, No	536	499	553	985	874	1003	4450
Referral rate, % (95% CI)	1.1 (1.0-1.2)	0.9 (0.9-1.0)	1.0 (1.0-1.1)	1.6 (1.5-1.7)	1.3 (1.2-1.4)	1.5 (1.4-1.6)	1.3 (1.2-1.3)
Screen detected breast cancers, No	224	274	254	345	321	353	1,771
Advanced cancers, No (rate*; 95% CI)	74 (1.5; 1.2-1.9)	87 (1.6; 1.3-2.0)	88 (1.6; 1.3-2.0)	99 (1.6; 1.3-1.9)	97 (1.5; 1.2-1.8)	125 (1.9; 1.5-2.2)	570 (1.6; 1.5-1.8)
Non-advanced cancers, No (rate*; 95% CI)	147 (3.0; 2.5-3.5)	178 (3.3; 2.8-3.8)	160 (3.0; 2.5-3.5)	239 (3.9; 3.4-4.4)	218 (3.3; 2.9-3.7)	222 (3.3; 2.9-3.7)	1,164 (3.3; 3.1-3.5)
Unknown tumor stage, No (rate*; 95% CI)	3 (0.1; 0.0-0.1)	9 (0.2; 0.1-0.3)	6 (0.1; 0.0-0.2)	7 (0.1; 0.0-0.2)	6 (0.1; 0.0-0.2)	6 (0.1; 0.0-0.2)	37 (0.1; 0.1-0.1)
Cancer detection rate* (95% CI)	4.6 (4.0-5.2)	5.1 (4.5-5.7)	4.7 (4.2-5.3)	5.6 (5.0-6.2)	4.8 (4.3-5.4)	5.2 (4.7-5.8)	5.1 (4.8-5.3)
PPV of referral, % (95% CI)	41.8 (37.6-46.0)	54.9 (50.8-59.5)	45.9 (41.9-50.2)	35.0 (32.0-38.0)	36.7 (33.5-39.9)	35.2 (32.1-38.0)	39.8 (38.3-41.2)
Interval cancers, No	75	94	128	116	139	117	669
Advanced cancers, No (rate*; 95% CI)	39 (0.8; 0.5-1.1)	63 (1.2; 0.9-1.5)	86 (1.6; 1.3-1.9)	79 (1.3; 1.0-1.6)	87 (1.3; 1.0-1.6)	70 (1.0; 0.8-1.3)	424 (1.2; 1.1-1.3)
Non-advanced cancers, No (rate*; 95% CI)	33 (0.7; 0.4-0.9)	30 (0.6; 0.4-0.8)	39 (0.7; 0.5-1.0)	35 (0.6; 0.4-0.8)	50 (0.8; 0.5-1.0)	46 (0.7; 0.5-0.9)	233 (0.7; 0.6-0.7)
Unknown tumor stage, No (rate*; 95% CI)	3 (0.1; 0.0-0.1)	1 (0.0; 0.0-0.1)	3 (0.1; 0.0-0.1)	2 (0.0; 0.0-0.1)	2 (0.0; 0.0-0.1)	1 (0.0; 0.0-0.0)	12 (0.0; 0.0-0.1)
Sensitivity, % (95% CI)	74.9 (70.3-80.1)	74.5 (70.0-78.9)	66.5 (61.8-71.2)	74.8 (70.9-78.8)	69.8 (65.6-74.0)	75.1 (72.0-79.8)	72.6 (71.0-74.5)
Proportion of advanced cancers among screen detected cancers + interval cancers, % (95% CI)	37.8 (32.4-43.4)	40.7 (36.3-46.3)	45.5 (40.0-50.0)	38.6 (34.2-43.1)	39.9 (35.7-44.7)	41.5 (37.0-46.0)	40.7 (38.8-42.7)

*per 1,000 women screened; CI = confidence interval; PPV = positive predictive value

Of the 2,679 false-positive referral, diagnostic work-up was limited to additional breast imaging in 60.6% (1,622/2,677) of women, whereas 29.6% (793/2677) underwent percutaneous biopsy (fine needle aspiration cytology of core needle biopsy) in addition. Excisional biopsy, with or without previous percutaneous biopsy, had been performed in 9.6% (256/2,677) of false-positive referrals.

### Advanced breast cancers detected at screening

Of 1,771 screen detected cancers, 570 were advanced cancers and 1,164 non advanced cancers. The tumor stages of the remaining 37 screen detected cancers could not be properly classified, including TxN- cancers (negative lymph nodes but unknown tumor size) and T1Nx cancers (invasive cancers ≤ 20 mm with unknown lymph node status); these were excluded from further analysis. A total of 290 cancers had been detected at an initial screening round, consisting of 105 advanced cancers (36.2%) and 185 non-advanced cancers. At subsequent screening, a total of 1,444 cancers had been detected, comprising 466 advanced cancers (32.3%) and 978 non-advanced cancers (36.2% versus 32.3%, p < 0.001). The proportion of advanced screen-detected cancers per 2-year screening period fluctuated between 28.7% (2003/2004) and 35.4% (2007/2008) (p = 0.6, Table 1). Univariate analysis showed no statistically significant differences between women with advanced or non-advanced breast cancer regarding family history of breast cancer, use of hormone replacement therapy, percentage initial screens, interval between screens, prior visibility or breast density (Table 2). Compared to non-advanced cancers, advanced screen detected cancers were more frequently characterized by abnormal densities and less frequently by suspicious microcalcifications at screening (p < 0.001) and comprised more invasive lobular cancers and fewer invasive ductal cancers (p < 0.001, Table 2).

**Table 2:** Characteristics of women with breast cancer

	Screen detected cancer			Interval cancer		
	Advanced N = 570	Non-advanced N = 1,164	P- value	Advanced N = 424	Non-advanced N = 233	P- value
Mean age, years (95%CI)	62.0 (61.3 - 62.6)	62.4 (62.0 - 62.8)	0.24	59.5 (58.9 - 60.2)	59.3 (58.4 - 60.2)	0.76
Family history of breast cancer^±^, No (%)	123 (21.6)	218 (18.7)	0.16	76 (17.9)	43 (18.5)	0.87
Previous breast surgery^#^	58 (10.0)	130 (11.2)	0.53	72 (15.6)	34 (15.0)	0.43
Use of hormone replacement therapy, No (%)	59 (10.4)	95 (8.2)	0.13	70 (13.1)	20 (6.3)	0.005
Initial screens, No (%)	106 (18.6)	183 (15.7)	0.13	64 (13.5)	32 (11.8)	0.64
Interval between 2 latest screens, No (%)			0.08	-	-	
< 30 months	527 (92.5)	1,101 (94.6)				
≥ 30 months	43 (7.5)	63 (5.4)				
Breast density at latest screening mammogram, No (%)			0.45			0.49
≤ 50%	375 (65.8)	787 (67.4)		243 (60.7)	140 (60.1)	
> 50%	195 (34.2)	377 (32.4)		181 (39.3)	93 (39.9)	
Mammographic abnormality, No (%)			< 0.001			0.04
Density	426 (74.8)	740 (63.6)		133 (64.6)	78 (70.9)	
Microcalcifications	35 (6.1)	280 (24.1)		26 (12.6)	18 (16.4)	
Density with microcalcifications	82 (14.4)	112 (9.6)		16 (7.8)	6 (5.5)	
Architectural distortion	22 (3.9)	24 (2.1)		11 (5.3)	7 (6.4)	
Breast parenchyma asymmetry	4 (0.9)	8 (0.7)		20 (9.7)	1 (0.9)	
Breast cancer visible at previous screening mammogram	199 (42.9)*	418 (42.7)^¥^	0.95	208 (47.6)	112 (47.4)	0.81
Tumor histology of invasive cancers, No (%)			< 0.001			0.09
Ductal	414 (72.6)	682 (77.7)		304 (71.8)	155 (77.7)	
Lobular	93 (16.3)	85 (9.7)		87 (21.3)	30 (10.9)	
Mixed ductal-lobular	37 (6.5)	41 (4.7)		18 (4.0)	10 (5.9)	
Invasive other	24 (4.2)	68 (7.7)		11 (2.0)	11 (5.5)	
Unknown	2 (0.4)	2 (0.2)		4 (0.9)	0 (0.0)	

^±^At least one first-degree relative (mother, sister, daughter) with a diagnosis of breast cancer before the age of 50 years or at least two second-degree relatives with breast cancer. ^#^Surgery for benign conditions (e.g., excisional biopsy, breast reduction, breast augmentation, mastitis) and malignant conditions (lumpectomy, mastectomy). *464 subsequent screens; ^¥^979 subsequent screens

After adjustment for all other variables (Table 3), we found that an interval of 30 months or more between the latest two screens was associated with increased risk of advanced screen detected breast cancer (OR 1.63, 95% CI: 1.07-2.48). High breast density was borderline significantly associated with increased risk of advanced breast cancer (OR 1.25, 95% CI: 0.99-1.57), as was a family history of breast cancer (OR 1.20, 95% CI: 0.93-1.56).

**Table 3:** Odds of having advanced breast cancer among women with breast cancer, each variable adjusted for all others

	Advanced vs. non-advanced screen detected cancers			Advanced vs. non-advanced interval cancers		
	OR	95%CI	P-value	OR	95%CI	P-value
Age						
50-59	1	0.57-0.95	0.02	1	0.80-2.48	0.2
60-69	0.73	0.61-1.12	0.2	1.41	0.33-1.52	0.4
70+	0.83			0.71		
Family history of breast cancer						
No	1	0.93-1.56	0.16	1	0.66-2.33	0.5
Yes	1.20			1.24		
Previous breast surgery						
No	1	0.64-1.26	0.5	1	0.41-2.04	0.8
Yes	0.90			0.90		
Use of hormone replacement therapy						
No	1	0.80-1.68	0.4	1	1.22-7.53	0.02
Yes	1.16			3.04		
Initial screen						
No	1	0.88-1.61	0.2	1	0.41-2.04	0.8
Yes	1.19			0.91		
Interval between 2 latest screens						
< 30 months	1			-		
≥ 30 months	1.63	1.07-2.48	0.02			
Breast density at latest screening mammogram						
≤ 50%	1			1		
> 50%	1.25	0.99-1.57	0.06	1.03	0.62-1.72	0.9
Mammographic abnormality						
Density	1			1		
Microcalcifications	0.20	0.14-0.29	0.0001	1.41	0.64-3.13	0.4
Density with microcalcifications	1.24	0.91-1.69	0.2	0.39	0.11-1.42	0.2
Architectural distortion	1.49	0.82-2.72	0.2	0.98	0.35-2.73	1.0
Breast parenchyma asymmetry	0.79	0.23-2.66	0.7	1.43	0.52-3.94	0.5
Tumor histology of invasive cancers^1^						
Ductal	1			1		
Lobular	2.00	1.44-2.78	0.0001	1.48	0.70-3.14	0.3
Mixed ductal-lobular	1.55	0.97-2.48	0.07	0.65	0.20-2.06	0.5
Invasive other	0.63	0.39-1.02	0.07	0.84	0.21-3.32	0.8
Unknown	1.70	0.23-12.3	0.6	-^2^		

1. Calculated for invasive cancers only. 2. Not calculated because all 4 patients with unknown histology had advanced breast cancer

**Figure 3:**
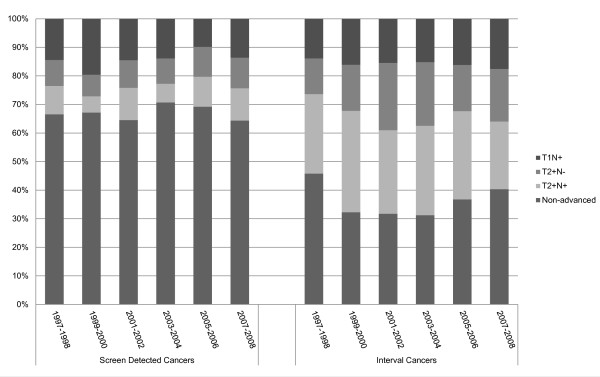
**Distribution of breast cancer stage at 6 consecutive 2-year screening periods**.

Of the 570 advanced screen-detected cancers, 106 (18.6%) had been detected at the initial screen and 464 (81.6%) at a subsequent screen. Of the latter, 265 (57.1%) were considered mammographically occult at the last but one screen at retrospect, whereas 88 (19.0%) showed a minimal sign and 111 (23.9%) were missed at the last but one screen. Thus, at least 65.1% (106+265/570) of advanced cancers could not have been diagnosed at an earlier stage. We observed no significant changes in the proportions of T1N+, T2+N- and T2+N+ screen detected cancers during our twelve-year screening period (Figure 3).

### Advanced interval breast cancers

Advanced breast cancers comprised 63.4% (424/669) of all interval cancers (Figure 1). Of advanced interval cancers, 35.8% (152/424) were diagnosed in the first year after the latest negative screen, and 64.2% (272/424) in the second year. Of interval cancers diagnosed in the first or the second year after the latest negative screen, respectively 65.0% (152/234, 95%CI: 58.8-72.1) and 62.5% (272/435, 95%CI: 60.0-67.1) were advanced cancers. At review, 50.9% (216/424) of advanced interval cancers were considered mammographically occult at the latest screen, whereas 103 (24.3%) showed a minimal sign and 105 (24.8%) had been missed. At univariate analysis, we found no significant difference between advanced and non-advanced interval cancers in family history of breast cancer, percentage initial screens, interval between screens, prior visibility, tumor histology or breast density (Table 2). Compared to the non-advanced cancers, advanced interval cancers were more frequently characterized by breast parenchymal asymmetries and less frequently by suspicious microcalcifications or abnormal densities (p = 0.04) and more women with advanced interval cancer used hormone replacement therapy (p = 0.005), (Table 2). The use of hormone replacement therapy was an independent risk factor for advanced breast cancer at multivariate analysis (OR 3.04, 95% CI: 1.22-7.53, Table 3). Similar to advanced screen detected cancers, we found no significant changes in the proportions of T1N+, T2+N- and T2+N+ interval cancers during twelve years of screening (Figure 3).

## Discussion

During twelve years of biennial screening, we did not observe a decline in advanced breast cancers. After review of previous mammograms, it had to be concluded that the majority of advanced breast cancers could not have been detected at an earlier tumor stage. Multivariate analysis showed that a screening interval of 30 months or more significantly increased the risk of detecting breast cancer in an advanced stage.

In a meta-analysis on randomized controlled mammography screening trials, Autier et al. calculated an equal decrease in breast cancer mortality for each unit decrease in incidence of advanced breast cancer [23]. We expected to find a reduction of advanced cancers in our screened population over time, as a result of increasing experience of the screening radiologists, continuous quality assurance, introduction of additional film reading by technologists and the increased use of 2-view mammography at subsequent screening mammography [16,24]. The incidence rate of advanced screen detected cancers and advanced interval cancers remained constant in our study and this finding is in line with a recent meta-analysis of regional and nation-wide screening programs, where annual percent changes in advanced breast cancer were stable or even increasing back to pre-screening rates [13]. As expected, the proportion of advanced breast cancers among screen detected cancers in our study was lower in subsequent screens, probably as a result of lead time.

The majority of advanced cancers in our biennial screening program of women aged 50-75 years could not have been detected earlier. More than half of advanced breast cancers detected at a subsequent screen were not visible at previous screening mammography. This high percentage may partly be due to our definition of advanced cancer, which included small (≤ 20 mm) but lymph node positive invasive cancers. Almost one-fifth of all advanced screen detected cancers had been discovered during the first screening round.

A previous study showed that the introduction of sentinel node biopsy in the Southeast region of The Netherlands had led to stage migration, as was reflected by an increased proportion of patients with positive axillary lymph nodes after adjustment for tumor size and age [25]. In order to prevent bias in our findings by this stage migration, we therefore analyzed our data using different definitions of advanced breast cancer. We found no decrease in advanced cancers over time, neither when advanced cancers were defined as invasive tumors exceeding 20 mm in size, nor when a definition of lymph node positive tumors was used for advanced cancers. Although introduction of the sentinel node technique has changed the diagnostic procedure for lymph node involvement, it is likely that determination of tumor size has remained constant over time and across institutions.

Almost 20% of our advanced screen-detected cancers showed a minimal sign at the previous screen. Earlier referral of these women may potentially decrease the proportion of advanced cancers. However, minimal signs were found to be present in 10% of screening mammograms, whereas less than 1% of these lesions turned out to be malignant [21]. The Dutch screening program would no longer be cost-effective if all these women are being referred [26]. Moreover, the maximum reduction in advanced cancers will probably be less than 20% as some minimal signs may be early signs of already advanced tumors, and thus will compromise the gain.

One quarter of advanced screen detected cancers had been missed at the previous screening examination and this proportion did not change significantly over time. In 2009, just after the end of our study, the two analogue screening units in the Southern Region were replaced by digital units. Moreover, independent double reading has been replaced by blinded double reading and all screening radiologists receive information about their individual screening performance at regular intervals. Full-field digital mammography has been shown to have similar or higher sensitivity and higher specificity than conventional mammographic screening and may ultimately lead to a decrease of advanced cancers detected at screening [27,28]. The introduction of digital screening in the Netherlands has resulted in increased referral rates and increased overall cancer detection rates [29,30]. The ultimate impact of all these changes on the future incidence of advanced cancers at screening mammography is not yet known.

At multivariate analysis, a prolonged screening interval was independently associated with advanced screen detected breast cancer and women aged 60-69 were also at risk of being diagnosed with advanced cancer. Further research on the reasons for skipping one or several screening rounds is needed in the effort to maximize screening adherence and thus minimize extended intervals between two screens. Cancers characterized by suspicious microcalcifications at screening were associated with a lower risk of advanced screen detected cancer, which can be explained by the fact that ductal carcinoma in-situ frequently shows microcalcifications as the only mammographic abnormality. This finding confirms the high sensitivity of mammography for the detection of calcium deposits that are typical of ductal, often in situ, non-dangerous breast cancers. Screening sensitivity is lower in dense breasts and, in our study, high breast density was borderline significantly associated with advanced cancer detected at screening. Postmenopausal women taking hormone replacement therapy are at increased risk of breast cancer [31,32] and, in our study, in women with interval cancers, hormone therapy was associated with an increased risk of having the cancer diagnosed at an advanced tumor stage. The association of hormone replacement therapy with advanced interval cancer, but not with advanced screen detected cancer, may reflect differences in tumor biology between advanced interval cancers and screen detected cancers and merits further investigation.

Tumor histology differed significantly between advanced cancers and early cancers detected at screening. Invasive lobular cancer was diagnosed more frequently in the advanced cancer group. Compared to invasive ductal cancers, invasive lobular cancers are more difficult to detect at mammography as these tumors more commonly present as subtle architectural distortions or focal asymmetric densities resembling that of normal breast parenchyma, or show no mammographic abnormalities at all [33].

The percentage of advanced cancers among women with interval breast cancer was almost twice the percentage of advanced cancers among women with screen detected cancer and also remained stable throughout our twelve-year screening period. Our observation that tumor stages of interval cancers were worse than those of screen detected cancers is expected and in line with previous reports [34,35]. Similar to advanced cancers detected at screening, half of the advanced interval cancers were mammographically occult at the latest screen and another quarter had been missed. Other studies also retrospectively classified 20-35% of all interval cancers as missed cancers [36,37]. Our observation of a similar proportion of advanced interval cancers among the total group of interval cancers diagnosed in the first and second year is in line with the findings reported by Porter et al [37]. The finding that a majority of both early stage and advanced interval cancers were diagnosed in the second year after the latest negative screen suggests that shortening of our screening interval may potentially lower the number of advanced interval cancers. For screen detected cancers however, two US studies found no increase in late-stage disease for women aged 50 years or older with a 2-year versus 1-year screening interval [38,39]. Moreover, annual screening will be more expensive and the concomitant larger numbers of false positive referrals probably increases patient anxiety and thus may have a negative impact on future screening adherence. For these reasons, investigators still argue about the optimal screening interval [40].

In the Netherlands, the incidence of breast cancer is still increasing, with a current lifetime risk of 13% [41]. Although screening may be effective in reducing breast cancer mortality, a possible future decrease in breast cancer mortality in screened women may rather be the result of advances in breast cancer treatment than the result of improved detection at screening mammography. Moreover, the rate of advanced cancer after implementation of screening mammography was comparable to pre-screening rates. Another detrimental effect of screening is the generation of false positive referrals, leading to increased levels of anxiety and additional diagnostic workup costs [42,43]. Although the positive predictive value of referral in our population was considerably higher than the one found in other European and US screening studies, 60% of referrals turned out to be false positive and almost 10% of false positively referred women had undergone excisional biopsy at diagnostic workup. Finally a potential harmful effect of screening is the phenomenon of so-called over-diagnosis of breast cancers, i.e. diagnosis of breast cancers that, if left undiscovered, would never become clinically evident and, thus, would never become lethal [44,45].

While non-advanced cancer rates have increased since the introduction of screening [20], we did not observe an expected decrease in advanced breast cancer rates. This finding may partly be explained by differences in tumor biology between non-advanced cancers and advanced cancers and our results suggest that screening may not be effective in detecting highly proliferative, aggressive breast cancers at an early stage. The stable rate of advanced cancers in our study also implies that a significant portion of breast cancers detected at screening represents over-diagnosis. A gradual increase in advanced breast cancer rate was observed in women below 50 years of age and this upward trend may reflect an underlying increase in background incidence of advanced disease.

Our study has several strengths and limitations. To our knowledge, this is the first study with virtually complete follow-up in which we determined the percentage of unavoidable advanced cancers at screening and assessed risk factors for advanced cancer at an individual level. Unfortunately, a stratified analysis of stage III and IV tumors was not possible due to low numbers of these cancers in our study. Furthermore, extrapolation of our results to other screening programs may be limited as the study designs of these programs show considerable variations. For example, the Dutch nation-wide screening program is characterized by a much lower referral rate than that of other screening programs. Moreover, screening outcome parameters will be influenced by the screening interval used at screening programs. Many European programs, including the Dutch one, offer biennial screening for women aged 50-75 years. In contrast, women are screened every 3 years in the UK and US programs often offer annual screening [2,46].

## Conclusion

In summary, we found no decline in advanced screen detected cancers and advanced interval cancers during twelve years of screening mammography and a majority of these advanced cancers could not be prevented at biennial screening. In order to obtain a modest reduction of advanced cancers detected a screening, efforts are needed to minimize extended screening intervals.

## Abbreviations

TNM: Tumour-Node-Metastasis Classification;

## Competing interests

The authors declare that they have no competing interests.

## Authors' contributions

JN collected data and drafted the manuscript. LD collected data, reviewed screening mammograms and participated in design of the study. AV performed the statistical analysis and helped to draft the manuscript. JG participated in the design of the study and performed the statistical analysis. FJ collected data, reviewed screening mammograms. ML performed the statistical analysis, conceived of the study, and participated in its design and coordination and helped to draft the manuscript. All authors read and approved the final manuscript.
